# Electric Bioimpedance Sensing for the Detection of Head and Neck Squamous Cell Carcinoma

**DOI:** 10.3390/diagnostics13142453

**Published:** 2023-07-24

**Authors:** Andrea Luigi Camillo Carobbio, Zhuoqi Cheng, Tomaso Gianiorio, Francesco Missale, Stefano Africano, Alessandro Ascoli, Marco Fragale, Marta Filauro, Filippo Marchi, Luca Guastini, Francesco Mora, Giampiero Parrinello, Frank Rikki Mauritz Canevari, Giorgio Peretti, Leonardo S. Mattos

**Affiliations:** 1 IRCCS Ospedale Policlinico San Martino, 16132 Genoa, Italy; a.carobbio@studenti.unibs.it (A.L.C.C.); stefano2africano@gmail.com (S.A.); alessandro.ascoli@outlook.it (A.A.); fragalemarco@yahoo.it (M.F.); mfilauro@yahoo.com (M.F.); filippomarchi@hotmail.it (F.M.); luca@guastini.eu (L.G.); francesco.mora@unige.it (F.M.); giampiero.parrinello@gmail.com (G.P.); canevari@edu.unige.it (F.R.M.C.); giorgioperetti18@gmail.com (G.P.); 2 Department of Surgical Sciences and Integrated Diagnostics (DISC), University of Genoa, 16132 Genoa, Italy; 3 Section of Otorhinolaryngology-Head and Neck Surgery, Department of Neurosciences, University of Padua-“Azienda Ospedaliera di Padova”, 35128 Padua, Italy; 4 Maersk Mc-Kinney Moller Institute, University of Southern Denmark, 5230 Odense, Denmark; zch@mmmi.sdu.dk; 5 Department of Advanced Robotics, Istituto Italiano di Tecnologia, 16163 Genova, Italy; tomaso.gianiorio@iit.it (T.G.); leonardo.demattos@iit.it (L.S.M.); 6 Department of Molecular and Translational Medicine, University of Brescia, 25125 Brescia, Italy; 7 Department of Head & Neck Oncology & Surgery, Antoni Van Leeuwenhoek, Nederlands Kanker Instituut, 1066 Amsterdam, The Netherlands; 8 Department of Experimental Medicine (DIMES), University of Genoa, 16132 Genoa, Italy

**Keywords:** squamous cell carcinoma, electrical bioimpedance, cancer detection, classifier, head and neck cancer, machine learning

## Abstract

The early detection of head and neck squamous cell carcinoma (HNSCC) is essential to improve patient prognosis and enable organ and function preservation treatments. The objective of this study is to assess the feasibility of using electrical bioimpedance (EBI) sensing technology to detect HNSCC tissue. A prospective study was carried out analyzing tissue from 46 patients undergoing surgery for HNSCC. The goal was the correct identification of pathologic tissue using a novel needle-based EBI sensing device and AI-based classifiers. Considering the data from the overall patient cohort, the system achieved accuracies between 0.67 and 0.93 when tested on tissues from the mucosa, skin, muscle, lymph node, and cartilage. Furthermore, when considering a patient-specific setting, the accuracy range increased to values between 0.82 and 0.95. This indicates that more reliable results may be achieved when considering a tissue-specific and patient-specific tissue assessment approach. Overall, this study shows that EBI sensing may be a reliable technology to distinguish pathologic from healthy tissue in the head and neck region. This observation supports the continuation of this research on the clinical use of EBI-based devices for early detection and margin assessment of HNSCC.

## 1. Introduction

Head and neck cancer is the sixth most common type of malignant tumor in the world, with more than 500,000 new cases annually [1]; it is responsible for an estimated 1–2% of all cancer deaths. Head and neck squamous cell carcinomas (HNSCCs) constitute more than 90% of head and neck cancers [2]. They encompass a heterogeneous group of malignancies that can arise from different subsites within the larynx, oral cavity, oropharynx, hypopharynx, and nasopharynx. Among these areas, those most frequently affected by SCC are the oral cavity (44%) and the larynx (31%). The management of HNSCC is focused on early detection, because delay in the diagnosis increases the incidence of regional and/or distant metastasis, leading to a very low 5-year survival rate [3]. This highlights the importance of incessant research and innovation, since any attempt to reduce the time of diagnosis is worth undertaking.

In recent decades, a number of optical and imaging technologies, such as bioendoscopy [4,5,6] and advanced radiologic examinations, have been investigated to enhance the clinical diagnostic accuracy. In addition, 3-dimensional evaluations of tumors are performed by combining endoscopy to define the superficial spread of the lesion and Magnetic Resonance Imaging (MRI) [7,8,9], Computerized Tomography (CT) [10], and Positron Emission Tomography (PET) [11] to measure its deep parts. However, these technologies are often limited by their high costs, long waiting lists, and the availability of properly trained personnel. At the completion of a diagnostic assessment for HNSCC, a biopsy is still mandatory for all suspicious lesions to obtain a definitive histopathological diagnosis.

Recently, the technological research field in cancer detection has been enriched by the introduction of Electrical Bioimpedance (EBI) sensing technology [12,13]. By exciting a tissue region with a controlled electrical signal, its electrical characteristics can be measured and used for tissue characterization. Malignant tumors have dissimilar electrical features compared to their healthy counterparts: this can be partially explained by their altered intracellular water and mineral content, membrane permeability, as well as the tissue cytoarchitecture and orientation of cells [14]. This has been demonstrated on various tissue types such as breast [15], prostate [16], liver [17,18] larynx [19], kidney [20], and oral cavity [21,22,23,24].

Based on these assumptions, this study aims to evaluate the electrical properties of cancerous and healthy tissues by measuring their EBI values, specifically in head and neck sites, which only a few researchers have explored to date [21,22,23]. Furthermore, we aim to present the use of the SmartProbe, a novel, compact, and cost-effective needle-based EBI sensor device for acquiring bioimpedance data and identifying cancerous tissue in HNSCC patients. 

## 2. Materials and Methods

### 2.1. Patients Selection

A prospective observational ex vivo study was conducted between May 2019 and October 2020 in the Otorhinolaryngology and Head and Neck Unit of the San Martino Polyclinic Hospital in Genoa, Italy. EBI data from tissue samples from 57 patients undergoing surgical procedures for head and neck tumors were collected with the SmartProbe system. The ex vivo experiments were conducted in accordance with the experimental protocol approved by the local Ethical Committee (Comitato Etico Regionale Liguria, study number 181/2018). 

### 2.2. SmartProbe Device

The SmartProbe system is designed as a needle-based bipolar EBI sensing system for clinical studies on the electrical characteristics of both pathologic and healthy tissues. Further technical details and evaluations of the SmartProbe can be found in previous papers [25,26]. The SmartProbe exploits a commercial, single-use, sterile concentric electrode needle (CEN, F8990/45, FIAB SpA, Firenze, Italy) to probe tissue. The central core of the needle and the needle tube itself are used as 2 electrodes, through which excitation signals are applied to the tissue and reciprocal signals are measured. According to Amini et al. [27], excitation frequencies from 10 kHz to 100 kHz are selected. The impedance value of a 0.4% saline solution is used as a reference [25], and the subsequently measured tissue impedances are projected to this reference to obtain the corresponding electrical conductivity (σ) and phase angle (θ). The SmartProbe system was certified to be in compliance with the CEI EN 60601-1 medical device standard by a third party. This confirmed that the prototype could be used in the operating room for studies on ex vivo tissue. 

### 2.3. Measurement Procedures

The SmartProbe device was installed in the operating room of the Unit of Otorhinolaryngology and Head and Neck surgery. Each EBI measurement was performed at the end of the resection procedure on the freshly excised ex vivo specimen within 15 min. To respect this timeline, the measurement executor was never involved in the surgical procedure. Once the specimen was available to be tested, the calibration procedure in saline solution was started with a new disposable CEN. Then, before starting the tissue measurements, information about the tissue specimen was recorded by the measurement executor through the graphic user interface (GUI). Specifically, the “Patient ID” field was filled out and the “Tissue Type” was selected from a predefined list (Supplementary Figure S1). In addition, useful notes about the tumor were collected, such as the macroscopic characteristics of the tissue or the exact location of the measurement site. 

For each measurement, the executor inserted the CEN into the tissue and checked the stability of the sensed data by observing the EBI polar plot displayed on the GUI. By pressing a button on the needle holder, impedance scanning with excitation frequency varying from 10 kHz to 100 kHz with 10 kHz steps was performed, and the respective EBI values were automatically saved in the laptop with the exact time of recording. The executor repeated the operation three times for every measurement site and then changed to the next site for measurement. Once the different tissue types from the specimen had been tested, the executor clicked the “Stop” button on the GUI to end the acquisition process, and the system automatically saved the data package to the laptop.

### 2.4. Matching Process with Histopathology

When the pathologist reported the definitive histopathology, every single measurement from each tissue type was systematically associated with a specific pathological status: “pathologic” for at least in situ carcinoma, “healthy” for normal tissue, “N/A” (Non-Assessable), i.e., for acquisitions in which a strictly objective relationship with the relative histopathology was not ascertainable. All these uncertain pieces of information were excluded from the statistical analysis (149 out of 2264 measurements, i.e., 6.58%). Only data coming from patients with a definitive histology of SCC were considered, and thus, all the non-SCC histotypes, benign lesions, and normal specimens were removed from the dataset (N = 11).

### 2.5. Data Analysis

Statistical significance tests were performed to investigate the differences between the EBI values acquired from pathologic and healthy tissues. This was performed separately for each of the five tissue types represented in the acquired dataset, i.e., cartilage, lymph node, mucosa, muscle, and skin. During the data analysis, the Kolmogorov-Smirnov test was used to verify the normality of the data, while the Kruskal-Wallis test was performed to compare the two conditions of each tissue type. Data were plotted with the use of Nyquist plots [28]. This analysis was repeated for every excitation frequency used to acquire the EBI measurements. The performance of the different algorithms was tested by the Holm method on the predicted results generated by the different algorithms.

In all analyses, a significance level of 5% was used. In this study, Matlab 2019b (MathWorks Inc., Natick, MA, USA) was used for statistical analyses, and the scikit learn library in Python was used for the design of classifiers.

### 2.6. Classifiers

Classifiers based on support vector machine (SVM) [29], XGBoost [30], and random forest [31] were developed to assess the potential for the automatic detection of cancer tissue based on the EBI measurements. This was done using the EBI data acquired from all different patients included in the study; these data were grouped into different sub-datasets according to the tissue type information. Then, the impedance data (including magnitude and phase at different frequencies) for each tissue type and each pathological status were grouped as the input and were given the same label. For each method, five classifiers were generated to distinguish between a healthy and pathologic status of the different tissue types. During the training of the SVM classification models, different kernel functions were tested, and the best one was found to be the Cubic polynomial. To evaluate the classifiers, a standard 5-fold cross-validation was used.

An additional investigation involved the development and assessment of patient-specific classifiers. For this, new sub-datasets were defined considering each single patient and each tissue type separately. Only data from patients for which both healthy and pathologic conditions were available for the specific tissue types were included in these sub-datasets. Then, different classifiers based on SVM, XGBoost, and random forest were developed and tested. 

The performance assessment of each developed classifier was based on standard metrics, including Sensitivity, Specificity, Accuracy, F1 score, Matthew’s Correlation Coefficient (MCC), and Area under the ROC Curve (AUC). 

## 3. Results

### 3.1. Clinico-Pathological Features of the Cohort

From a cohort of 57 patients recruited for the study, 46 with a histopathological diagnosis of SCC were enrolled in the study. Eleven patients were excluded from the analysis due to a pathologic report different from SCC. Among the included patients, 3 were subsequently excluded due to technical problems in the calibration and/or acquisition process that generated inconsistent data. Finally, 43 patients were included, with a total of 2015 EBI datapoints available for the statistical analysis. A summary of the data collected for each tissue is shown in Table 1. Note that each EBI data consists of 10 different EBI values acquired by the impedance scanning with the excitation frequency changing from 10 kHz to 100 kHz.

### 3.2. Tissue-Specific Data

**Figure 1:**
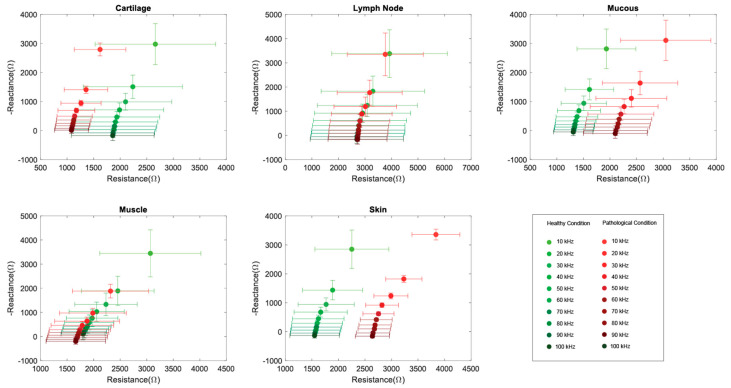
Tissue-specific data: Nyquist plots with 2D error bars summarizing the data from all patients included in the study. The mean and SD of Resistance and Reactance are shown for each of the 10 frequencies (from 10 kHz to 100 kHz).

For each tissue type, a Nyquist plot was generated to show the change of the real part (resistance) and imaginary part (reactance) as a function of the excitation frequency (Figure 1). Standard descriptive statistics was used for data summaries and to express means and standard deviations. The results of our measurements on healthy and pathologic tissues were plotted in green and red, respectively. For all tissue types, both the resistance and reactance were higher at low excitation frequencies and decreased as the frequency increased (Supplementary Figure S2). Statistical significance was investigated between the pathologic and healthy condition data for each of the five tissue types. The Kolmogorov-Smirnov test was used to verify the normality of the different datasets, while the Kruskal Wallis test was performed to compare the datasets of healthy and pathologic tissues. This analysis was repeated for every frequency, and the results are summarized in Table 2.

### 3.3. Performance of the Tissue-Specific Classifiers

**Figure 2:**
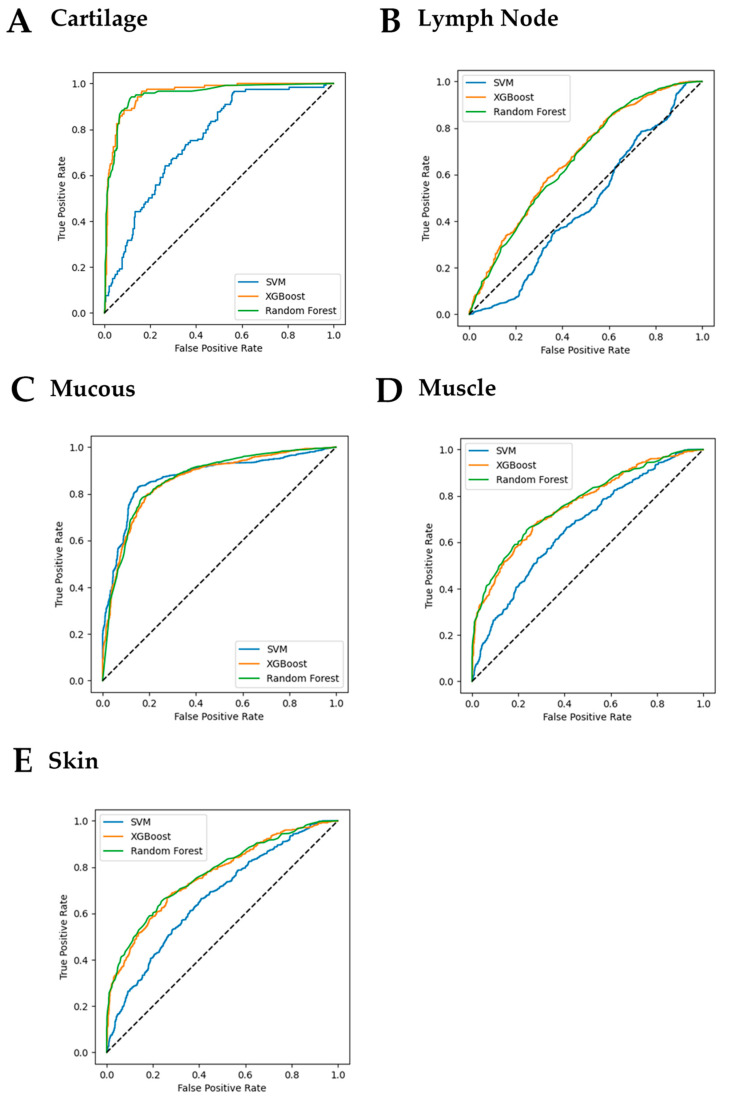
Receiver operating characteristic (ROC) curves showing the performance of the support vector machine (SVM), XGBoost, and Random Forest models (by five-fold cross-validation).

The obtained tissue-specific performance metrics are summarized in Table 3. The ROC curve (receiver operating characteristic curve) for different training models can be found in Figure 2. 

The tissue-specific classifiers developed based on data from all patients achieved considerably high sensitivity in distinguishing pathologic tissue from healthy tissue. The best overall results were achieved on skin tissue, demonstrating sensitivity, specificity, and accuracy values above 0.92. 

The data in Table 3 shows that, for mucosa tissues, the best results were achieved with the SVM classifier. However, for other tissues, both the XGBoost and the Random Forest classifiers tended to achieved performances superior to those of the SVM classifiers. Nonetheless, when using the Holm’s method to compare the overall performance of the classifiers based on the predicted values, the results ruled out a statistically significant difference in their performance. Table 4 reports the results of this analysis. The ROC curves in Figure 2, on the other hand, highlight the superiority of the XGBoost and the Random Forest classifiers over SVM. 

### 3.4. Patient-Specific Data

During data collection, it often happened that only healthy or pathologic specimens of a tissue type were available for EBI measurement. Therefore, the amount of data in the patient-specific sub-datasets was variable, as shown in Table 1. Nonetheless, the results from the data analysis of each patient-specific sub-dataset are described below and in Table 5.

- **Mucosa**: For 10 out of 13 patients, the SVM classifiers achieved 1.00 sensitivity and specificity. For the other 3 patients, considerably high sensitivity (0.85, 0.93, and 0.97) and specificity (0.93, 0.79 and 0.97) were also achieved. The accuracies of 12 classifiers were higher than 0.87, while one classifier achieved an accuracy of only 0.54. In addition, we found that for 3 out of 13 patients, the differences in the bioimpedance of healthy mucosa and SCC mucosa were not statistically significant. However, for the other 10 patients, the differences were statistically significant (*p*-values < 0.001). For the results based on random forest classifiers, only data from four subjects achieved 1.00 sensitivity and specificity. Nevertheless, the classification accuracy was generally high, given that data from 9 patients were higher than 0.90, from 2 patients were between 0.80 to 0.90 and from the other 2 patients were between 0.75 to 0.80. The results of the classification using the XGBoost method revealed that data from 2 patients achieved 1.00 accuracy, data from 8 patients achieved an accuracy ranging from 0.90 to 1.00, and data from another 3 patients achieved an accuracy between 0.80 and 0.90.
- **Lymph node:** The analysis here included only one patient for whom data from both healthy and pathologic lymph nodes were available. The data of healthy specimens and pathologic specimens were found to have statistically significant differences. The accuracies achieved by the developed SVM, XGBoost, and Random Forest classifiers were found to be 0.79, 0.81 and 0.82 respectively. Statistical analysis (Kruskal-Wallis test) on the data revealed that the values from pathological lymph node tissue were significantly different from those obtained from healthy tissues, given a *p*-value < 0.001.
- **Muscle:** In this case, only one patient was included in the analysis. Both the SVM classifier and the Random Forest classifier achieved 0.93 accuracy, while a slightly higher accuracy, i.e., 0.95, was achieved by the XGBoost classifier. Statistical analysis also proved that the bioimpedance of healthy muscle tissue was significantly different from that of pathologic muscle tissue.
- **Cartilage:** Data from two patients were available for this analysis. The SVM classifier for one of them achieved 1.00 accuracy and 0.63 accuracy for the other patient. The classification accuracies achieved by the XGBoost classifier and the Random Forest classifier were relatively lower, but all were higher than 0.75. Significant differences were found between healthy tissue data and pathologic tissue data for both patients’ data (*p*-value < 0.001).
- **Skin:** In this case, data from three patients were included in the analysis, and accuracies of 1.00, 0.99 and 0.72 were achieved by the developed SVMs. The accuracies by XGBoost were found to be 1.00, 0.98 and 0.84. When the Random Forest classifier was used, the accuracies were reported to be 1.00, 0.98 and 0.86. The *p*-values of the statistical analysis between healthy and pathologic data were found *p* < 0.001, *p* = 0.006, and *p* = 0.051.

## 4. Discussion

The results of this study regarding the use of electrical bioimpedance sensing to detect head and neck cancer confirm that the technology can distinguish healthy from malignant tissue. As observed, the electrical impedance of biological tissues is complex and varies with the excitation frequency. Resistance arises mainly from the interaction of extra and intercellular fluids for different types of cells and tissues [32]. In contrast, the capacitive reactance depends on the characteristics of cellular membranes and the frequency of the applied signal [33]. At low frequency, the magnitude of the capacitive reactance is relatively large, which impedes the current from passing through the cell membrane, leading it to flow instead through the extracellular space among the cells. In this case, the tissue resistivity depends upon the cell spacing, intercellular bonds, and tissue arrangement. When the excitation frequency increases, the membrane capacitive reactance gradually decreases and the current starts to pass through the cells. Therefore, EBI values measured using high frequency signals are also determined by cellular volume and intracellular characteristics, like the dimensions of the nucleus [33,34]. Potentially, a wider sensing spectrum could improve cancerous tissue detection. For instance, the results of healthy and pathologic muscle tissues overlap more in the higher frequency range, whereas at lower frequencies, the measurements are better separated, as shown in the Nyquist plots. In fact, the data in Supplementary Table S1 show that when considering only the EBI values acquired at 10 kHz for tissue-specific classification, both the SVM and the Random Forest classifiers performed slightly better than when the full frequency range was used. This provides an interesting insight to transform the SmartProbe into a real-time tissue classification system, as it can work at 140 Hz when measuring EBI at a single excitation frequency. 

Literature data indicate that different tissues have different electrical properties as a consequence of their different cellular salt content, the altered packing density of cells, and the different layout of the tissue [35]. Nevertheless, tissue heterogeneity may present a challenge for tissue identification, since it can lead to variations in impedance measurements [36,37]. Even for the same tissue type and status, every measured region may contain a slightly different ratio of intra-cellular and extra-cellular components [38]. This is the case also when comparing the same tissue type from different patients. For this reason, it is challenging to create a single classification model that performs well on data from all patients. To guarantee high levels of performance, the tissue classification model should generalize well and be robust against noisy signals. Achieving this would require much more data to better train the classifiers. In addition, other AI methods (e.g., deep learning) could be investigated for tissue classification, which may perform better than the classification methods tested in this work.

It is interesting to see that according to the results of patient-specific investigations, cancer detection accuracy can be improved considering a patient-specific rather than a tumor-based approach. For instance, this was seen when analyzing the data from all tissue types, which allowed us to create classifiers with higher accuracy than the classifiers developed based on the overall tissue-specific datasets. This indicates that the different physiological parameters of different people might influence the electrical properties of the tissues, and thus, patient-specific tissue classification may be a way to achieve a reliable and robust bioimpedance-based cancer detection system. According to this finding, not only a larger amount of data, but also a higher number of patients should be included in future analyses.

A change of impedance values of biological tissues after cell death has been reported in other studies [39,40]. To minimize the risk of measurement biases in this paper, data acquisition was always performed within 15 min of specimen resection. Nonetheless, the collected EBI values could still be different from those from in vivo tissues, which may contain noise from physiological activities and static charges [41]. 

The ex vivo results obtained herein endorse several new applications of this new diagnostic technique. Particularly in early stage tumors, histologic parameters in preoperative biopsy specimens may not represent the whole resection specimen, like in oral cavity SCCs [42], sinonasal tumors [43], or laryngeal leukoplakias [44]. Additionally, biopsy samples can only represent a small portion of the lesion, and thus, the accurate selection of the sampling site is important, as unrepresentative tissue samples can lead to misdiagnoses, treatment delays, and/or possible undertreatments. The use of an electrical bioimpedance sensing device such as the SmartProbe, particularly if combined with up-to-date bioendoscopy technologies during in vivo clinical practice, could be beneficial to detect the most representative spot in a suspicious superficial lesion and to obtain a more reliable tumor sampling during the incisional biopsy. The feasibility of EBI measurements during in vivo assessments of oral cavity lesions has already been described by other authors in the literature [21,22,23,24].

Furthermore, the same technique may be applied in an intra-operative setting to help surgeons in establishing the extension of the surgical resection and to check the surgical margins. A similar concept and intraoperative setting has been developed to distinguish brain tumor tissues from surrounding healthy tissues before and during the surgical resection [45].

Another possible future application may be to combine the EBI with ultrasound-guided fine-needle aspiration cytology, which is mainly applied for the diagnosis of suspect cervical lymph nodes [46], salivary glands nodules, and thyroid nodules [47], guided by preoperative imaging. Despite being often associated with high values of sensitivity and positive predictive values, its reported sensitivity and negative predictive value are still modest, with a false negative rate exceeding 30% in some series regarding cervical lymph node evaluations [48,49,50]. Similar results were obtained in the evaluation of suspicious thyroid nodules, with a non-diagnostic result of up to 30%, as reported in a recent meta-analysis [47].

## 5. Conclusions

EBI is a proven method for the detection of cancer in different sites. The current literature also describes its possible role in head and neck cancer assessments, particularly for oral cavity diseases. Nevertheless, the diffusion of this technique is still far from being wide. In this paper, EBI measurements were accomplished by the use of the SmartProbe, a new, needle-based device that proved to be a useful and effective tool for quick, simple, and low-cost EBI data acquisition on fresh ex vivo tissue samples. The results of this study corroborate the assessment of EBI as a helpful technology to distinguish cancer from healthy tissue, especially since statistically significant differences were observed for four different tissue types (mucosa, muscle, skin, and cartilage). In addition, it was noted that the use of patient-specific data greatly improved the sensitivity, specificity, and accuracy of the cancer detection algorithms. This study is still ongoing in our hospital, but these preliminary results already provide strong support for the development of a clinical device for in vivo practice. This is expected to be a possible complementary tool for target biopsies, FNACs, and intraoperative resection margin evaluations, flanking the currently available technologies and enhancing their diagnostic capabilities.

## Figures and Tables

**Table 1:** Number of EBI data collected for each tissue type.

Type	Overall (N)	Healthy Tissue N (%)	Pathologic Tissue N (%)	n° of Patients (Total)	n° of Patients (*)
Cartilage	162	115 (71%)	47 (29%)	11	2
Lymph Node	763	491 (64%)	272 (36%)	25	1
Mucous	525	141 (27%)	384 (73%)	28	13
Muscle	421	261 (62%)	160 (38%)	32	1
Skin	144	82 (57%)	62 (43%)	9	3

* Number of patients involved in the patient-specific study for whom EBI data from both healthy and pathologic tissue were available.

**Table 2:** Summary of the Kruskal Wallis test results (*p*-values) comparing the difference between the EBI data from pathological and healthy tissue samples for different excitation frequencies.

Tissue Type	Excitation Frequency (kHz)									
	10	20	30	40	50	60	70	80	90	100
Mucosa	<0.001	<0.001	<0.001	<0.001	<0.001	<0.001	<0.001	<0.001	<0.001	<0.001
Lymph node	0.44	0.19	0.19	0.62	0.49	0.15	0.13	0.21	0.48	0.09
Muscle	<0.001	<0.001	<0.001	<0.001	<0.001	<0.001	<0.001	<0.001	<0.001	<0.001
Cartilage	0.016	0.021	<0.001	<0.001	0.0157	0.012	0.009	0.046	0.141	0.001
Skin	<0.001	<0.001	<0.001	<0.001	<0.001	<0.001	<0.001	<0.001	<0.001	<0.001

**Table 3:** Performance metrics of tissue-specific classifiers developed based on data from the overall patient population (with five-fold cross-validation). The highest values are shown in bold. Legend: SVM, support vector machine; MCC, Matthew’s Correlation Coefficient; AUC, Area under the ROC Curve.

Performance Metric	Classifier	Mucosa	Lymph Node	Muscle	Cartilage	Skin
Sensitivity	SVM	0.86	**0.99**	0.20	0.42	0.91
	XGBoost	**0.87**	0.81	**0.58**	**0.76**	**0.95**
	Random Forest	0.86	0.79	**0.58**	0.72	0.94
Specificity	SVM	**0.76**	0.07	**0.93**	**0.98**	0.86
	XGBoost	0.70	0.43	0.81	0.95	0.91
	Random Forest	0.71	**0.44**	0.83	0.95	**0.92**
Accuracy	SVM	**0.83**	0.65	0.65	0.82	0.89
	XGBoost	0.81	**0.67**	0.72	**0.92**	**0.93**
	Random Forest	0.82	**0.67**	**0.73**	0.91	**0.93**
F1 score	SVM	**0.87**	**0.78**	0.30	**0.79**	0.90
	XGBoost	0.86	0.76	**0.62**	0.77	**0.94**
	Random Forest	0.86	0.75	**0.62**	0.74	**0.94**
MCC	SVM	**0.61**	0.16	0.19	0.46	0.77
	XGBoost	0.56	**0.26**	0.40	**0.72**	**0.86**
	Random Forest	0.57	0.25	**0.42**	0.68	**0.86**
AUC	SVM	**0.81**	0.53	0.56	0.52	0.88
	XGBoost	0.78	**0.62**	0.69	**0.86**	**0.93**
	Random Forest	0.79	**0.62**	**0.70**	0.83	**0.93**

**Table 4:** *p*-values of the performance pairwise comparisons between the tested algorithms by Holm’s method.

Classifier	Mucosa	Lymph Node	Muscle	Cartilage	Skin
SVM vs. XGBoost	1.0	1.0	0.74	0.93	1.0
SVM vs. Random Forest	1.0	1.0	0.74	0.93	1.0
XGBoost vs. Random Forest	1.0	0.76	0.74	0.056	1.0

**Table 5:** Average performance metrics for the patient-specific classifiers by five-fold cross-validation (shown as mean ± standard deviation). The highest values are shown in bold. Values for lymph node and muscle do not include standard deviation because the respective data are from a single patient.

Performance Metric	Classifier	Mucosa	Lymph Node	Muscle	Cartilage	Skin
Sensitivity	SVM	**0.95 ± 0.12**	0.24	**1.00**	0.80 ± 0.21	0.93 ± 0.09
	XGBoost	0.93.5 ± 0.09	**0.91**	0.94	0.73 ± 0.07	**0.97 ± 0.04**
	Random Forest	0.88 ± 0.26	0.88	0.95	**0.83 ± 0.17**	0.93 ± 0.08
Specificity	SVM	**0.94 ± 0.14**	**0.96**	0.90	0.86 ± 0.14	0.88 ± 0.17
	XGBoost	0.85 ± 0.21	0.52	**0.98**	**0.92 ± 0.83**	0.92 ± 0.09
	Random Forest	0.85 ± 0.26	0.63	0.90	0.88 ± 0.86	**0.96 ± 0.04**
Accuracy	SVM	**0.94 ± 0.13**	0.79	0.93	0.82 ± 0.18	0.90 ± 0.13
	XGBoost	0.91 ± 0.13	0.81	**0.95**	**0.87 ± 0.10**	0.94 ± 0.07
	Random Forest	0.87 ± 0.26	**0.82**	0.93	0.86 ± 0.11	**0.95 ± 0.06**

## Data Availability

The dataset is uploaded on Mendeley Dataset and publicly available. Missale, Francesco (2021), “Electric_Bioimpedance_H&N_Dataset”, Mendeley Data, V1, doi: 10.17632/b9t274zxgz.1.

## Supplementary Materials

The following supporting information can be downloaded at: https://www.mdpi.com/article/10.3390/diagnostics13142453/s1, Supplementary figures, Figure S1: The SmartProbe system Graphic User Interface (GUI) showing (A) a system status window; (B) a polar plot with a red dot indicating the impedance value measured at 100 KHz; (C) system mode selection buttons; (D1) optional “note” field and (D2) two obligatory information fields to be filled for every patient; (E) multiple-choice menus to select the calibration method and the tissues characteristics. Figure S2: Bode plots for each tissue type. Table S1: Performance metrics of tissue-specific classifiers based on 10 kHz impedance values from the overall patient population (with 5-fold cross-validation). Legend: SVM, support vector machine; F1, F1 score; MCC, Matthew’s Correlation Coefficient; AUC, Area under the ROC Curve.
