# Supplementary material for: Electric Bioimpedance Sensing for the Detection of Head and Neck Squamous Cell Carcinoma
Source: Diagnostics (Basel). 2023 Jul 24;13(14):2453. doi: 10.3390/diagnostics13142453 (PMC10377945; doi:10.3390/diagnostics13142453)
Supplement: Supplementary file 1 [file diagnostics-13-02453-s001.zip › diagnostics-2423933-supplementary.pdf]

Electric bioimpedance sensing for the detection of head and neck squamous cell carcinoma: a preliminary feasibility study

Supplementary Material

Supplementary Figures

Figure S1: The SmartProbe system Graphic User Interface (GUI) showing A) a system status window; B) a polar plot with a red dot indicating the impedance value measured at 100 KHz; C) system mode selection buttons; D_1) optional "note" field and D_2) two obligatory information fields to be filled for every patient; E) multiple-choice menus to select the calibration method and the tissues characteristics.

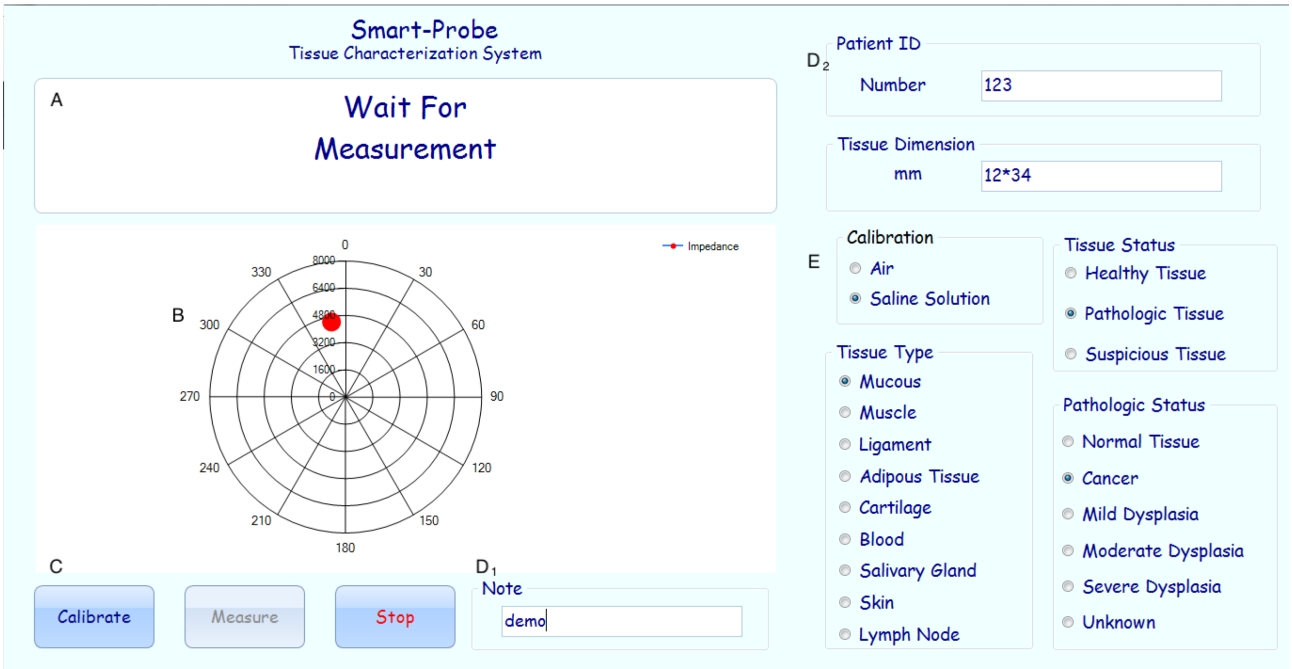

Figure S2: Bode plots for each tissue type. In red are represented the results of the pathological samples and in green the healthy ones.

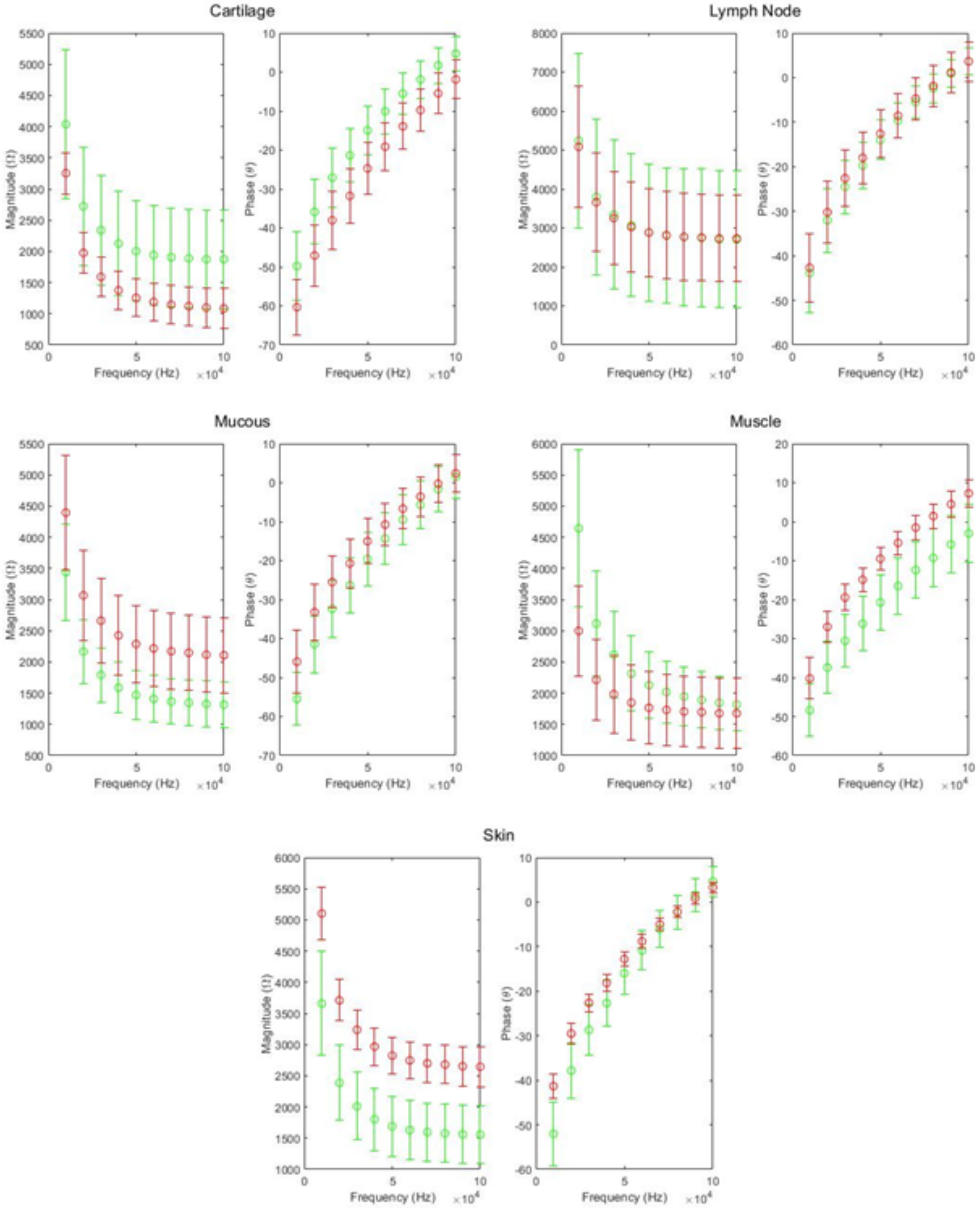

Table S1: Performance metrics of tissue-specific classifiers based on 10 kHz impedance values from the overall patient population (with 5-fold cross-validation). Legend: SVM, support vector machine; F1, F1 score; MCC, Matthew's Correlation Coefficient; AUC, Area under the ROC Curve.

Method	Type	Mucosa	Lymph Node	Muscle	Cartilage	Skin
SVM	**Sensitivity**	0.85	0.73	0.78	0.92	0.89
	**Specificity**	0.78	0.50	0.96	1.00	0.95
	**Accuracy**	0.83	0.64	0.87	0.99	0.92
	**F1**	0.88	0.70	0.85	0.96	0.93
	**MCC**	0.59	0.24	0.75	0.95	0.83
	**AUC**	0.87	0.67	0.95	1.00	0.99
XGBoost	**Sensitivity**	0.89	0.74	0.92	0.67	0.96
	**Specificity**	0.77	0.51	0.91	0.93	0.95
	**Accuracy**	0.85	0.66	0.91	0.88	0.96
	**F1**	0.89	0.73	0.89	0.67	0.96
	**MCC**	0.66	0.25	0.82	0.59	0.91
	**AUC**	0.83	0.62	0.91	0.80	0.96
Random Forest	**Sensitivity**	0.90	0.72	0.93	0.75	0.93
	**Specificity**	0.74	0.51	0.90	0.94	1.00
	**Accuracy**	0.85	0.64	0.91	0.91	0.96
	**F1**	0.89	0.72	0.88	0.75	0.96
	**MCC**	0.65	0.22	0.81	0.69	0.92
	**AUC**	0.87	0.70	0.97	0.94	0.96
